# Prevalence of Congenital Cytomegalovirus Infection in a CMV-Endemic Ugandan Population

**DOI:** 10.1093/ofid/ofag566

**Published:** 2026-09-05

**Authors:** Amy K F Davis, Mary K Prahl, Mikias Ilala, Justine Levan, Pamela M Odorizzi, Mary Muhindo, Abel Kakuru, Stephanie L Gaw, Moses R Kamya, Grant Dorsey, Margaret E Feeney

**Affiliations:** Department of Pediatrics, University of California, San Francisco, California, USA; Department of Pediatrics, University of California, San Francisco, California, USA; Department of Medicine, University of California, San Francisco, California, USA; Department of Medicine, University of California, San Francisco, California, USA; Department of Medicine, University of California, San Francisco, California, USA; Infectious Diseases Research Collaboration, Kampala, Uganda; Infectious Diseases Research Collaboration, Kampala, Uganda; Department of Obstetrics, Gynecology & Reproductive Sciences, University of California, San Francisco, California, USA; Infectious Diseases Research Collaboration, Kampala, Uganda; Department of Medicine, Makerere University, Kampala, Uganda; Department of Medicine, University of California, San Francisco, California, USA; Department of Pediatrics, University of California, San Francisco, California, USA; Department of Medicine, University of California, San Francisco, California, USA

**Keywords:** congenital infection, cytomegalovirus, global health, maternal-fetal health

## Abstract

Congenital cytomegalovirus (cCMV) infection is a leading cause of childhood deafness and disability worldwide. However, less is known about its epidemiology in low-income regions where most women are CMV-seropositive at conception. We found that cCMV is highly prevalent in rural Uganda (3.73%) and is associated with lower gravidity and placental malaria. We additionally report a high transmission intensity in the first years of life, with 97.3% of children acquiring CMV IgG by age 3.

Globally, cytomegalovirus (CMV) is the most common congenital infection and causes substantial infant morbidity, including prematurity, fetal growth restriction, microcephaly, developmental delay, and sensorineural hearing loss (SNHL). Approximately 10%–15% of infants with congenital CMV (cCMV) are symptomatic at birth, and another 10% develop delayed sequelae such as SNHL [1]. As such, congenital CMV is the leading cause of nongenetic hearing loss [2] and a major cause of poor neurodevelopmental outcomes in children.

CMV is ubiquitous worldwide, but there is wide geographic variation in its seroprevalence. Among adults, the prevalence of CMV infection ranges from ∼20% in some high-income countries to nearly 100% in many low-income countries [3]. CMV immunity prior to pregnancy has been shown to influence the risk of congenital transmission. Women who experience primary infection with CMV during pregnancy have a very high risk of vertical transmission (30%–40%), whereas women who are already CMV-seropositive prior to conception may still transmit the virus to their infants (termed “non-primary infection”), but the risk of transmission is much lower (1%–2%). Congenital CMV occurs in ∼0.6% of pregnancies in high-income countries [4], where approximately half of women are CMV-seropositive at conception. The epidemiology, incidence, and determinants of congenital CMV in many low-income countries remain poorly characterized, but current data suggest that the prevalence of congenital CMV may, in many such regions, exceed that of high-income countries [5–7].

Here, we examine the prevalence of congenital CMV infection in a birth cohort of Ugandan infants and demonstrate a very high rate of transmission in this highly CMV-endemic population. We also evaluate maternal epidemiologic factors associated with vertical CMV transmission, including age, gravidity, and maternal coinfection with other pathogens. Furthermore, we report on the clinical sequelae of congenital CMV and the rates of early childhood acquisition of CMV in this population. Our results demonstrate that even in populations with widespread early-life CMV acquisition (and therefore high maternal preconception immunity), congenital CMV remains prevalent and is associated with significant clinical sequelae.

## METHODS

### Patient Consent Statement

Cord blood samples were obtained from infants enrolled in a clinical trial of malaria chemoprevention in pregnancy and early childhood conducted in Eastern Uganda (PROMOTE-BC1: NCT02163447, PROMOTE-BC2: NCT02282293, PROMOTE-BC3: NCT02793622) [8–10]. Mothers were enrolled at 12–20 weeks gestation, and children were followed up to 3 years of life. Written informed consent was obtained from the mothers of all study participants. The study protocol was approved by Makerere University School of Biomedical Sciences Ethics Committee, Uganda National Council of Science and Technology, and the University of California, San Francisco, Research Ethics Committee.

### cCMV Screening

DNA from 1046 cord blood samples was extracted using the QIAmp Blood Mini Kit (Qiagen). Screening for CMV DNA was performed by quantitative reverse transcription polymerase chain reaction (RT-qPCR) using PowerUp™ SYBR™ Green Master Mix (Applied Biosystems™) on the ABI StepOnePlus system. All samples were screened in duplicate using UL123 forward (TCCCGCTTATCCTCRGGTACA) and reverse (TGAGCCTTTCGAGGASATGAA) primers. Positive samples were confirmed with RT-qPCR using UL55 forward (TGGGCGAGGACAACGAA) and reverse (TGAGGCTGGGAAGCTGACAT) primers. All samples with discordant replicates were rescreened by digital droplet polymerase chain reaction (ddPCR) for the final call. For ddPCR, the same UL55 primer set was used along with a UL55 probe (TGGGCAACCACCGCACTGAGG). After droplet emulsion using the Bio-Rad QX200, PCR and analysis were performed on the Bio-Rad ddPCR system.

For RT-qPCR, a consistent exponential amplification across technical replicates and a melt curve matching the positive control were used to call positives. For ddPCR, ≥3 droplets per well were considered positive.

### Statistical Analysis

Statistical analysis was performed using STATA 15.1. Statistical significance was assigned when a *P*-value <.05 was obtained. Univariate analysis was used to calculate relative risk. Significant variables in univariate modeling were included in multivariate binomial regression to calculate adjusted relative risk.

### Pediatric CMV Screening

Longitudinal plasma samples from a subset of 260 infants were assessed for the acquisition of CMV-specific IgG by ELISA (Diamedix Immunosimplicity®-IS CMV IgG) during the first 3 years of life. Fifty-six-week, 108-week, and 156-week samples were screened. A total of 260 donors, including 12 with cCMV, were screened at 56 weeks; 2 children were lost to follow-up at 108 weeks, and 3 additional children were lost to follow-up at 156 weeks.

## RESULTS

### Prevalence of cCMV Infection in Uganda

A total of 1282 pregnant women were enrolled in parallel clinical trials comparing differing antimalarial regimens for the prevention of malaria in pregnancy in Uganda [8–10]. A total of 1046 cord blood samples were collected and screened for CMV DNA, and 3.73% (39/1046) of samples were positive. In our cohort, there was a significant association between maternal gravidity and cCMV infection, with an increased risk of cCMV transmission in primigravid mothers (risk ratio [RR] 2.39, 95% CI 1.14–5.00, *P* = .021) (Table 1). There was no association between maternal age or infant sex and cCMV (Table 1).

**Table 1.** Risk Factors Associated With Congenital Cytomegalovirus Infection

Variable	Categories	Congenital CMV n/N (%)	Univariate RR (95% CI)	*P V*alue	Multivariate RR (95% CI)	*P V*alue
Gravidity	Multigravida	13/552 (2.36%)	Reference	…	Reference	…
	Secundigravida	12/243 (4.94%)	2.10 (0.97–4.53)	.059	1.81 (0.82–3.98)	.141
	Primigravida	14/249 (5.62%)	2.39 (1.14–5.00)	.021	1.68 (0.73–3.84)	.223
Maternal age (years)	32–45	4/162 (2.47%)	Reference	…	Not included	…
	24–31	12/365 (3.29%)	1.33 (0.44–4.07)	.615	…	…
	16–23	23/517 (4.45%)	1.80 (0.63–5.13)	.270	…	…
Malaria placental histopathology positive	No	17/674 (2.52%)	Reference	…	Reference	…
	Yes	22/364 (6.04%)	2.40 (1.29–4.45)	.006	1.97 (0.98–3.95)	.056
HIV-positive	No	29/860 (3.37%)	Reference	…	Not included	…
	Yes	10/186 (5.38%)	1.59 (0.79–3.21)	.192	…	…
Birth of female infant	No	18/520 (3.46%)	Reference	…	Not included	…
	Yes	20/524 (3.82%)	1.10 (0.59–2.06)	.759	…	…

We assessed the impact of cCMV infection on infant outcomes. Among cCMV+ infants, 13.2% had microcephaly (<3%ile, n = 5), which was significantly associated with cCMV (RR 2.88, 95% CI 1.21–6.84, *P* = .017) (Table 2). A total of 21.1% of cCMV infants were born small for gestational age (SGA) (<3%ile; n = 8); this was also significantly associated with cCMV status (RR 2.86, 95% CI 1.49–5.50, *P* = .002). There was no association of cCMV with the risk of premature birth.

**Table 2.** Associations Between Congenital Cytomegalovirus Infection and Adverse Birth Outcomes

Sequelae	CMV-Positive Infants n/N (%)	CMV-Negative Infants n/N (%)	Relative Risk (95% CI)	*P V*alue
Premature (<37 weeks)	2/38 (5.26%)^a^	65/1005 (6.47%)	0.81 (0.21–3.20)	.768
Microcephaly				
<3%ile	5/38 (13.2%)^a^	45/985 (4.57%)	2.88 (1.21–6.84)	.017
<10%ile	9/38 (23.7%)^a^	124/985 (12.6%)	1.88 (1.04–3.41)	.037
SGA				
<3%ile	8/38 (21.1%)^a^	74/1006 (7.36%)	2.86 (1.49–5.50)	.002
<10%ile	11/38 (28.9%)^a^	189/1006 (18.8%)	1.54 (0.92–2.58)	.100

^a^One infant with congenital CMV was a stillbirth; birth measurements were not collected.

### Maternal Coinfection

We examined the impact of maternal coinfections on vertical transmission of CMV. Placental malaria (PM) is extremely common in eastern Uganda, and histopathologic examination of the placenta revealed evidence of PM in 35.2% (364/1038) of study participants from whom placental samples were obtained. Placental malaria was associated with an increased risk of cCMV (RR 2.40, 95% CI 1.29–4.45, *P* = .006) (Table 1). This association may be partly attributable to lower gravidity among mothers with placental malaria (adjusted risk ratio 1.97, 95% CI 0.98–3.94, *P* = .056). We additionally examined associations with the quantitative burden of placental malaria, as measured by the percentage of high-powered fields (HPF) in which malaria pigment in fibrin was present on placental histopathology. We found that a higher burden of malaria pigment deposition (≥10% of HPF) was associated with a further increased risk of cCMV (RR 4.03, 95% CI 1.16–14.03, *P* = .029) compared to cases without placental malaria.

All study participants were screened for HIV-1 upon enrollment at 12–20 weeks gestation, and 15.6% (200/1282) of women were HIV-positive. Of these, 106 of 188 women were viremic at study entry (HIV RNA >20 copies/mL). All participants with HIV who were not already receiving antiretroviral therapy (ART) initiated therapy upon enrollment. At delivery, 43 of 183 women had detectable HIV viremia. Overall, the risk of cCMV did not significantly differ between HIV-exposed and unexposed infants (RR 1.59, 95% CI 0.79–3.21, *P* = .192) (Table 1). However, there was an increased risk of cCMV in women with CD4 counts <200 (RR 7.76, 95% CI 2.48–24.30, *P* < .001) and viral loads ≥1000 copies/mL (RR 5.62, 95% CI 1.51–20.92, *P* = .010) at enrollment. Neither HIV RNA levels ≥1000 copies/mL nor CD4 count <200 at delivery were associated with risk of cCMV.

### High Rates of CMV Acquisition in Early Life

To better understand the timing of CMV acquisition in this epidemiologic setting, we assessed for the acquisition of CMV antibodies during the first 3 years of life in a subset of infants. We found that 222 of 260 (85.4%) children had CMV-specific IgG at 56 weeks of age, including 12 children with cCMV. At 108 weeks of age, 241 of 258 (93.4%) children were CMV-seropositive. A total of 248 of 255 (97.3%) children were CMV-seropositive at 156 weeks of age.

## DISCUSSION

Congenital CMV is a leading cause of adverse neurodevelopmental outcomes in childhood. In many African countries, the overwhelming majority of women are CMV-positive, but little is known regarding the epidemiology and risk factors for vertical CMV transmission in these highly endemic areas. Here, we report a high (3.73%) prevalence of congenital CMV infection among Ugandan infants, which is 5–10-fold higher than most high-income countries. Sequelae of congenital CMV, such as microcephaly and SGA, were observed in a similar proportion of cCMV-infected infants as have been reported in high-income countries, in accord with studies demonstrating that the mode of maternal infection (primary vs non-primary) does not impact the severity of congenital CMV [11]. Together, these data indicate that there may be a vast and silent epidemic of childhood disability due to *in utero* CMV infection among infants in low-income countries that has gone largely unstudied.

It is well established that women who are CMV-seropositive before pregnancy have a lower risk of vertical transmission than women who acquire primary CMV infection during pregnancy, suggesting that preexisting maternal immunity is partially protective against vertical transmission. Our data demonstrate a high rate of CMV acquisition in Uganda in the first years of life, indicating that most mothers have longstanding latent CMV infection. It is thus noteworthy that the prevalence of congenital CMV infection in Uganda far exceeds that seen in high-income countries (∼0.6%), where many reproductive-age women are CMV-seronegative and thus vulnerable to primary infection during pregnancy. However, this finding is supported by studies in Africa [5, 6] and other low-income countries [7] that reported similarly high prevalence of congenital CMV. This, coupled with studies showing that seropositive young women are commonly reinfected with new strains of CMV [12], raises the possibility that vertical transmission of CMV may in many cases result from superinfection of CMV-positive individuals with new, antigenically distinct viral strains. Further studies are needed to determine the relative contribution of reinfection or reactivation of latent maternal virus to congenital CMV transmission and whether this varies across epidemiologic settings. An alternate explanation for the high risk of transmission in this region is the impact of placental inflammation or immune compromise from endemic maternal coinfections.

We identified maternal factors that were associated with congenital CMV transmission, including concomitant maternal infections such as placental malaria. In our study, histologically confirmed placental malaria was associated with congenital CMV transmission; this is paralleled by a study in West Africa that demonstrated a similar association [6]. In a multivariate model examining gravidity and placental malaria, placental malaria approached but did not remain significant. This suggests that this association may be partially attributable to gravidity, owing to the propensity of placental malaria to occur in first and second pregnancies [13]. Placental malaria causes marked placental inflammation, so it remains reasonable to postulate that this inflammation may affect transplacental viral spread [13]; this is further supported by our observation that transmission risk correlated with the extent of histopathological inflammation. We additionally studied congenital CMV transmission in women with HIV. Maternal HIV status was not associated with congenital CMV in our cohort; however, this may partly reflect that all women with HIV were on or initiated ART at enrollment. Notably, mothers with HIV who transmitted CMV had significantly higher viral loads and lower CD4 counts at enrollment than nontransmitting mothers, which is in agreement with prior reports that poorly controlled maternal HIV infection increases the risk of vertical CMV transmission [14].

Our findings suggest that the risk factors for vertical transmission of CMV may differ across epidemiologic settings. In high-income populations, younger age and higher parity are independently associated with increased risk of congenital CMV infection [15]. This is believed to be due in part to the risk of primary maternal CMV infection among mothers caring for young children, who frequently acquire CMV in group childcare settings. In our study, maternal age was not associated with transmission risk. In contrast to data from high-income countries, we observed that mothers of infants with congenital CMV had *lower* average gravidity than nontransmitting mothers, with the highest risk in primigravidas. Two other studies in highly CMV-endemic populations also found primiparity [6] and lower gravidity [16] to be associated with increased risk of congenital CMV transmission. It is possible that the differences in risk factors among populations relate to differences in the predominant routes of CMV transmission to pregnant women (eg, household transmission from young children vs sexual transmission) [17].

Our study had several limitations. While congenital CMV was associated with SGA and microcephaly, other typical sequelae of congenital CMV, like rash, hepatomegaly, and SNHL, were not assessed. CMV diagnosis was based on PCR of whole cord blood, which could miss infected infants whose viremia resolved prior to delivery; we did not have access to samples of infant saliva or urine, which could improve sensitivity in such cases. Nonetheless, we believe this work is a meaningful step toward understanding the epidemiology of congenital CMV in low-income countries.

Our findings highlight the prevalence of congenital CMV in Africa and the need to develop new preventive strategies to protect infants from this devastating infection. Existing cCMV-preventive strategies exclusively target seronegative women who are at risk of developing primary CMV infection during pregnancy, even though primary infection accounts for only one-fourth of the congenital CMV cases in the U.S. and an even smaller fraction globally. A better understanding of the maternal immune determinants of vertical transmission is urgently needed to guide the development of CMV vaccines and prophylactic strategies for use in pregnancy. Interrupting the vertical transmission of CMV globally could avert almost a million cases of neurodevelopmental disability yearly and may improve outcomes from other childhood infections like tuberculosis and HIV, where CMV can worsen disease progression.
